# Use of Electronic Ecological Momentary Assessment Methodologies in Physical Activity, Sedentary Behavior, and Sleep Research in Young Adults: Systematic Review

**DOI:** 10.2196/46783

**Published:** 2023-06-29

**Authors:** Kimberly R Hartson, Luz Huntington-Moskos, Clara G Sears, Gina Genova, Cara Mathis, Wessly Ford, Ryan E Rhodes

**Affiliations:** 1 School of Nursing University of Louisville Louisville, KY United States; 2 Christina Lee Brown Envirome Institute University of Louisville Louisville, KY United States; 3 Kornhauser Health Sciences Library University of Louisville Louisville, KY United States; 4 School of Exercise Science, Physical and Health Education University of Victoria Victoria, BC Canada

**Keywords:** ecological momentary assessment, young adults, 24-hour movement behaviors, physical activity, sedentary behavior, sleep, mobile phone

## Abstract

**Background:**

Recent technological advances allow for the repeated sampling of real-time data in natural settings using electronic ecological momentary assessment (eEMA). These advances are particularly meaningful for investigating physical activity, sedentary behavior, and sleep in young adults who are in a critical life stage for the development of healthy lifestyle behaviors.

**Objective:**

This study aims to describe the use of eEMA methodologies in physical activity, sedentary behavior, and sleep research in young adults.

**Methods:**

The PubMed, CINAHL, PsycINFO, Embase, and Web of Science electronic databases were searched through August 2022. Inclusion criteria were use of eEMA; sample of young adults aged 18 to 25 years; at least 1 measurement of physical activity, sedentary behavior, or sleep; English language; and a peer-reviewed report of original research. Study reports were excluded if they were abstracts, protocols, or reviews. The risk of bias assessment was conducted using the National Heart, Lung, and Blood Institute’s Quality Assessment Tool for Observational Cohort and Cross-Sectional Studies. Screening, data extraction, and risk of bias assessments were conducted by independent authors, with discrepancies resolved by consensus. Descriptive statistics and narrative synthesis were used to identify overarching patterns within the following categories guided by the Checklist for Reporting Ecological Momentary Assessments Studies: study characteristics, outcomes and measures, eEMA procedures, and compliance.

**Results:**

The search resulted in 1221 citations with a final sample of 37 reports describing 35 unique studies. Most reports (28/37, 76%) were published in the last 5 years (2017-2022), used observational designs (35/37, 95%), consisted of samples of college students or apprentices (28/35, 80%), and were conducted in the United States (22/37, 60%). The sample sizes ranged from 14 to 1584 young adults. Physical activity was measured more frequently (28/37, 76%) than sleep (16/37, 43%) or sedentary behavior (4/37, 11%). Of the 37 studies, 11 (30%) reports included 2 movement behaviors and no reports included 3 movement behaviors. eEMA was frequently used to measure potential correlates of movement behaviors, such as emotional states or feelings (25/37, 68%), cognitive processes (7/37, 19%), and contextual factors (9/37, 24%). There was wide variability in the implementation and reporting of eEMA procedures, measures, missing data, analysis, and compliance.

**Conclusions:**

The use of eEMA methodologies in physical activity, sedentary behavior, and sleep research in young adults has greatly increased in recent years; however, reports continue to lack standardized reporting of features unique to the eEMA methodology. Additional areas in need of future research include the use of eEMA with more diverse populations and the incorporation of all 3 movement behaviors within a 24-hour period. The findings are intended to assist investigators in the design, implementation, and reporting of physical activity, sedentary behavior, and sleep research using eEMA in young adults.

**Trial Registration:**

PROSPERO CRD42021279156; https://www.crd.york.ac.uk/prospero/display_record.php?ID=CRD42021279156

## Introduction

### Background

Young adults often have a reputation as “healthy”; however, there is a sharp decline in cardiovascular health during this life stage characterized by high levels of undetected and subclinical disease progression that leads to diagnosable chronic illness in middle adulthood [1]. Young adults also report poorer mental health and higher stress than other age groups [2]. There is substantial evidence that modifiable lifestyle behaviors, specifically physical activity, sedentary behavior, and sleep, can substantially decrease the risk of chronic illnesses such as mental illnesses [3-6] and cardiovascular diseases [7-10]. Thus, young adulthood, defined as the age range of 18 to 25 years [11], is a critical time for the development of healthy lifestyle behaviors to prevent chronic diseases.

Young adults struggle with developing healthy routines related to physical activity, sedentary behavior, and sleep. In 2019, only 27.7% of young adults in the United States met the physical activity recommendations for health [12]. In 2018, a systematic review [13] found that on average young adults in college spent 10 to 11 hours in sedentary activity per day, vastly exceeding the recommended maximum limit of 8 hours of sedentary behavior per day [14-16]. The COVID-19 pandemic has only served to worsen the problem in young adults, reporting decreases in physical activity and increases in sedentary behavior during this time [17,18]. Young adults also report high rates (30%-58%) of sleep difficulties [19-21]. Despite the high prevalence of unhealthy behavior patterns during this life stage, young adults have historically been underrepresented in behavioral intervention research compared with pediatric and older populations [1,22].

Movement behaviors (physical activity, sedentary behavior, and sleep) have traditionally been studied independently, without focusing on their interactions. In recent years, there has been a shift toward acknowledging and examining physical activity, sedentary behavior, and sleep duration together within a 24-hour period. There is an intuitive relationship among these constructs in that a 24-hour period is a fixed block of time; thus, if sleep duration is reduced, those hours are naturally reallocated to either physical activity or sedentary behavior time. There is increasing evidence that viewing these behaviors together within a 24-hour period has important implications for health [23-25]. In 2016, Canada developed the first 24-hour movement public health guidelines for children and youth [26], followed by 24-hour movement guidelines for adults and older adults in 2020 [16]. Several other public health agencies, including the World Health Organization, have followed suit with combined movement guidelines, particularly for younger populations [27-29]. There is increasing evidence that meeting 24-hour movement guidelines is associated with better health than meeting individual guidelines for physical activity, sedentary behavior, or sleep duration [27].

Recent technological advances in electronic ecological momentary assessments (eEMA) have increased the ease with which movement behavior data can be collected. Ecological momentary assessment (EMA) is a methodological approach that uses repeated sampling of participants for data collection to provide a real-time context. Therefore, eEMA uses electronic devices to administer frequent periodic surveys to participants. This sampling approach supports the validity of self-reported behaviors, allowing for a decreased impact of recall bias [30] that comes with the traditional use of surveys with longer recall durations (eg, 7-day physical activity recall). Thus, the eEMA methodology allows for the assessment of behaviors and related factors that fluctuate within individuals over time [31]. Owing to the dynamic nature and natural shifting of physical activity, sedentary behavior, and sleep within a 24-hour period, these behaviors are likely best assessed in a dynamic engagement context that includes frequent assessments collected in the participant’s natural environment. Furthermore, given the recent technological advances, this can be done using devices (eg, smartphones and web-enabled devices) that participants are often carrying daily [32]. Thus, data collection via eEMA provides an avenue to better understand the context and patterns of behaviors over time in a participant’s natural environment, making it a useful methodology for studying physical activity, sedentary behavior, and sleep.

In recent years, the scientific literature has seen a marked increase in studies that incorporate EMA methodology. A quick search completed in the PubMed database with the term “ecological momentary assessment” yields nearly 3500 citations; more than half of the studies were published between 2020 and 2022. Recent systematic reviews of EMA methodology have been completed in the areas of sedentary behavior or physical activity, with the most recent review including a search conducted in 2018 [33-35]. Despite the influx of original research, no reviews of EMA methodology in movement research included recent research (after 2018) or a focus on young adult populations.

### Objectives

The purpose of this systematic review was to describe the use of the eEMA methodology in physical activity, sedentary behavior, and sleep research among young adults. The research question guiding this systematic review is, “How is eEMA methodology used/implemented in physical activity, sedentary behavior, and sleep research with young adults?” This systematic review stands apart and extends available reviews in 4 ways: (1) we focused solely on the young adult age range of 18 to 25 years [11]; (2) we incorporated physical activity, sedentary behavior, and sleep; (3) we included studies through August 2022, which encompasses the COVID-19 era studies (2020-2022); and (4) we focused on the use of eEMA, thus acknowledging the advances in technology that have shaped the collection of real-time data. Therefore, this approach integrates updated concepts and technological advances to guide the precise and practical implementation of the eEMA methodology in the study of physical activity, sedentary behavior, and sleep in young adults.

## Methods

The protocol and reporting of this systematic review were guided by the PRISMA (Preferred Reporting Items for Systematic Reviews and Meta-Analyses) guidelines [36] and are registered with PROSPERO (CRD42021279156).

### Inclusion and Exclusion Criteria

The eligibility criteria are presented in Table 1. The inclusion criteria for a young adult sample were designed to allow for small variations in the definition of young adults while maintaining the focus on those aged 18-25 years.

**Table 1:** Eligibility criteria.

Domain	Included	Excluded
Article types	Peer-reviewed original research	Abstracts, protocols, meta-analyses, and reviews
Data collection	Use of eEMA^a^ to collect data for at least 1 construct (eg, a movement behavior or a correlate of a movement behavior); eEMA was defined as frequent repeated prompting (greater than once per day) via an electronic device (eg, phone, mobile app, text messaging, smartwatch, and web based) to collect self-reported data in real time in the participant’s natural environment	No eEMA (eg, use of only reflective daily diaries or recall surveys without frequent repeated prompting, collected in a research laboratory, or using paper and pencil only)
Sample	Age range 18-25 years; mean age 18-23 years and age range 17-29 years; or a sample described as “young adults” or “college students” if no age range or mean age was provided	Not young adult sample
Outcomes	Inclusion of at least 1 measure of physical activity (eg, duration or frequency), sedentary behavior (eg, duration or frequency), or sleep (eg, duration or quality)	Did not include a measure of physical activity, sedentary behavior, or sleep
Language	English	Not English

^a^eEMA: electronic ecological momentary assessment.

### Search Strategy

Systematic searches related to the use of eEMA in research on physical activity, sedentary behavior, or sleep in young adults were conducted in August 2021 by a librarian. A follow-up search using the same procedures was conducted in August 2022. The search included the following databases: PubMed, CINAHL, PsycINFO, Embase, and Web of Science. Limiters included English language, and no other limiters or filters were used. EndNote 20 (Clarivate) was used to manage the citations. Key search terms included the following with related terms and synonyms: “physical activity,” “sedentary behavior,” “sleep,” “ecological momentary assessment,” and “young adults.” Multimedia Appendix 1 provides the full search strategy.

### Study Selection Process

The clinical librarian (GG) conducted the search, removed duplicates, and compiled the citations with abstracts into Rayyan–a web-based application for systematic reviews (Rayyan) [37]. During the first round of screening, 2 authors independently reviewed each title and abstract (KRH and CM or LHM and WF). Manuscripts were included and excluded based on standardized eligibility criteria, with conflicts resolved by team discussion. During round 2, a total of 2 authors independently read each remaining full text (KRH and LHM), screening the articles against eligibility criteria with conflicts resolved by discussion. The references of the included articles were then screened for any relevant articles that may have been missed during the search.

### Risk of Bias Assessment

The risk of bias was assessed by 2 independent authors (KRH and RER) using the National Heart, Lung, and Blood Institute (NHLBI) Quality Assessment Tool for Observational Cohort and Cross-Sectional Studies [38]. This quality assessment instrument contains 14 items covering topics including adequately detailed reporting of research questions, study population, eligibility criteria, sample size justification or power analysis, valid and reliable measures, prospective and repeated measures, blinding, and confounding variables. Additional items included a participation rate of at least 50%, uniform application of criteria, recruitment from the same or similar populations (including time), adequate time frame for the study purpose, evaluation of different levels of exposure, and ≤20% loss to follow-up after baseline [38]. Each item was scored as yes (1), no (0), or other (0; cannot determine, not reported, and not applicable). Per the instrument guidelines, items marked as “no” or “other” counted for 0 points, whereas items marked as “yes” received 1 point. Discrepancies (intraclass correlation coefficient=0.86) were reconciled through discussion. An overall quality score was based on a sum of item scores with good quality meaning low risk for bias (scores 11-14), fair quality meaning moderate risk for bias (scores 5-10), and poor quality meaning high risk for bias (scores 0-4) [39].

### Data Extraction and Analysis

The data extraction process was developed collaboratively by the research team. The extracted categories and items were based on the Checklist for Reporting EMA Studies, an adapted Strengthening the Reporting of Observational Studies in Epidemiology checklist developed by Liao et al [40]. Data were extracted within the following categories: (1) study characteristics (eg, design, sample, demographics, and location); (2) measures and outcomes (eg, measurement of physical activity, sedentary behavior, or sleep; eEMA-measured outcomes; psychometric support; analysis; and temporality); (3) eEMA procedures (eg, incentives, technology, training, monitoring period, prompting design, prompting frequency, and prompting modality); and (4) compliance, attrition, and missing data. Definitions for these categories were based on those described by Liao et al [40]; for example, attrition was defined as a description of participation across days or waves of monitoring throughout the study, rather than a simple pre-post calculation of attrition. The data for each manuscript were independently extracted by 2 authors (KRH and LHM or KRH and CGS) using a standardized template based on the abovementioned categories. Discrepancies in data extraction were discussed until consensus was reached. If consensus could not be reached, a third author was included in the resolution.

Coding was developed through an iterative process by the first 3 authors (KRH, LHM, and CGS) based on the current eEMA literature [33-35,40,41] and emerging data from the reviewed reports. Discrepancies in coding were discussed until consensus was reached. Data were tabulated within each category. Data analysis included descriptive statistics (eg, frequency, range, and mean) of reported values and narrative synthesis to identify overarching patterns based on recurrence and repetition within the previously identified categories.

## Results

### Overview

**Figure 1:**
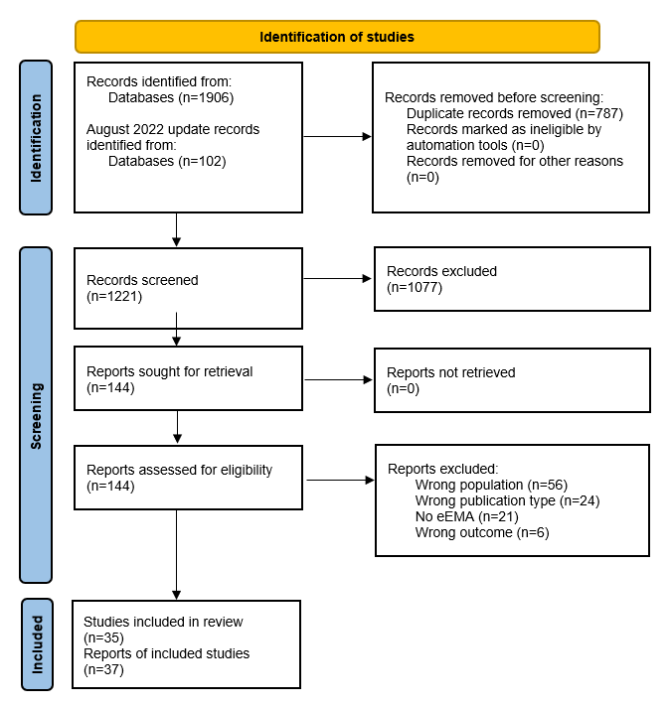
Study selection process. eEMA: electronic ecological momentary assessment.

The established search protocol yielded 1221 citations with duplicates removed. After title and abstract screening, 144 records remained for full-text screening. Of the 144 full-text records, 107 (74.3%) records were excluded for (1) not including a sample of young adults (n=56, 52.3%); (2) publication type (n=24, 22.4%); (3) not using eEMA (n=21, 19.6%); or (4) not including a physical activity, sedentary behavior, or sleep measure (n=6, 5.6%). No additional eligible studies were identified during the hand search of reference lists. This systematic process resulted in 37 eligible reports from 35 studies. Figure 1 shows an overview of the study selection process using a PRISMA flow diagram [36].

### Characteristics of Included Studies

Of the 37 reports included in this review, 18 (49%) were published between 2020 and 2022 and 10 (27%) were published between 2017 and 2019 (Table 2). Observational designs were used in 95% (35/37) of the reports, and 11% (4/37) of the reports had experimental components. Sample sizes ranged from 14 to 1584 young adults, with 28 (80%) of the 35 samples recruited from college students or apprentice populations. The other 7 samples either did not report the percentage currently enrolled in college or were not college-based samples. Six samples were subsamples from larger studies [42-47]. Most of the samples (20/35, 57%) consisted of multiple sexes or genders; a few samples consisted of female or women participants only (2/35, 6%) or men only (1/35, 3%). Sex or gender was not reported in 2 samples (5.7%). Racial or ethnicity demographics were reported in 23 (62%) of the 37 reports. The most reported racial demographic was White or European American (23/37, 62%); sample proportions range from 4% to 84%. Other commonly reported demographic categories included Black or African American (18/37, 49%; proportions range: 2.4%-100%); Asian (15/37, 41%; proportions range: 4.3%-84.7%); Hispanic, Latinx, or Spanish (12/37, 32%; proportions range: 2.4%-37%); and multiracial (11/37, 28%; proportions range: 3.7%-11.8%). Geographic locations varied across reports, with most studies conducted in the United States (22/37, 60%) and fewer conducted in Germany (4/37, 11%), Canada (2/37, 5%), the United Kingdom (2/37, 5%), Japan (2/37, 5%), Brazil (1/37, 3%), China (1/37, 3%), and Australia (1/37, 3%). Table 2 presents study characteristics.

**Table 2:** Study characteristics.

Study	Sample size, n	Age (years), mean (SD; range)	Sex or gender	Race or ethnicity	Location	Movement behaviors
Andorko et al [48],^a^ 2019	39 university students	20.22 (1.59; 18-25)	56% female	38% Asian, 23% Black or African American, 28% White, 8% multiracial, 3% other, and 3% Hispanic	United States	PA^b^^,c,d^ and SB^b,c,e^
Bedard et al [49],^a^ 2017	86 first-year university students	18.3 (0.51; —^f^)	53% female and 47% male	30% Asian, 7% South Asian, 6% Middle Eastern, 4% Black, 2% Indigenous, and 4% multiracial	Canada	PA^g^
Bernstein et al [50],^a,h^ 2019	76 undergraduate students	19.80 (1.24; 18-22)	70% female and 29% male	42.11% White, 7.89% African American, 34.21% Asian or Asian American, 2.63% Native American or American Indian, 11.84% multiracial, and 5.26% Hispanic or Latinx	United States	PA^b^
Bruening et al [42],^a^ 2016	41 university students	18.72 (0.50; —)	27% male	51% White, 10% Black, 12% multiracial or other, and 27% Hispanic	United States	PA^b^ and SB^b^
Burke et al [51],^a^ 2022	119 undergraduate students	19.87 (1.75; 18-26)	89.1% female	63.9% White, 15.1% Asian, 6.7% Black, 5% multiracial, 8.4% other, 0.8% preferred not to answer, and 8.4% Hispanic	United States	Sleep^c^^,^^g^
Das-Friebel et al [52],^a^ 2020	101 undergraduate students	19.70 (1.09; 18-22)	65.3% female	—	The United Kingdom	Sleep^c,g^
Gilchrist et al [53],^a^ 2021	89 undergraduate students	20.39 (1.59; 18-29)	53% female	75% White	United States	PA^b^
Kim et al [54],^a^ 2015	22 undergraduate students	21.9 (2.6; —)	90.9% male and 9.1% female	—	Japan	PA^g^
Kono et al [55],^a^ 2022	83 undergraduate students	20.24 (1.37; —)	60.2% female	—	Japan	PA^b^, sleep^b^
MacIntyre et al [56],^a^ 2020	74 undergraduate students	20.4 (1.63; 18-25)	100% women	59.5% Black, 41.9% White, and 12.2% other races	United States	PA^c,g^
Maher et al [57],^a^ 2020	50 first-year university students	(—; 18-19)	70% female	100% Black; 4% White; 2% another racial group; and 18% Hispanic, Latinx, or Spanish	United States	PA^g^
Maher et al [58],^a^ 2022	Same sample as Maher et al [58]	Same sample as Maher et al [57]	Same sample as Maher et al [57]	Same sample as Maher et al [57]	United States	PA^g^ and sleep^c,g^
Marquet et al [43],^a^ 2017	47 university students; subsample from Marquetet al [59]	19.5 (2.1; —)	49% female and 51% male	70% White, 21% Asian, and 9% other	United States	PA^b,c,g^
Marquet et al [59],^a^ 2018	74 university students	19.6 (—)	50% female and 50% male	77% White	United States	PA^b,c,g^
Mead and Irish [60],^a^ 2022	79 university students	19.01 (1.16; 18-25)	58.2% female and 41.8% male	83.5% White, 5.1% Black, and 11.4% Asian	United States	Sleep^c,g^
Miller et al [61],^k^ 2004	83 first-year university students	18.3 (0.9; —)	55.4% female	66.3% White, 24.10% Asian, 2.4% African American, 4.8% other, and 2.4% Hispanic	United States	PA^c^ and sleep^c^
Milyavskaya et al [62],^a^ 2018 (study 1)	159 first-year university students	18.0 (1.04; —)	72% female	—	Canada	PA^b^ and sleep^b^
Nadell et al [44],^a^ 2015	188 young adults	21.32 (—)	53.2% female	61.7% White, 13.3% Black, 4.3% Asian or Pacific Islander, 4.3% other, and 16.5% Hispanic	United States	PA^c^
Parsons et al [63],^a^ 2022	101 young adults	21.69 (1.91; 18-24)	84.2% female and 15.8% male	—	United Kingdom	Sleep^c^
Ponnada et al [45],^a^ 2022	Pilot: 15 young adults; main study: 81 young adults	Pilot: —; 18-24; main study: 21.7 (2.4; —)	45% female and 55% male	54% White, 46% Asian or Pacific Islander, 9% Black, 7% Native Indian or Alaska Native, and 37% Hispanic	United States	PA^b,g^ and SB^b,g^
Romanzini et al [64],^a^ 2019	126 young adults	—; 18-25	—	—	Brazil	SB^b,g^
Runyan et al [65],^a,h^ 2013	81 first-semester undergraduate students	18.26 (0.49; —)	56.8% female and 43.2% male	93.8% White	United States	PA^b^^,^^c^
Sala et al [66],^a^ 2017	129 university students	19.19 (1.40; 17-23)	100% female	51.2% European American, 26.4% Asian, 10.1% multiracial, 5.4% Black, and 6.2% Hispanic	United States	PA^b^
Sano et al [67],^a^ 2018	201 university students	—; 18-25	35.8% female and 64.2% male	—	United States	PA^b,g^ and sleep^b,g^
Shah et al [68],^a^ 2021	14 young adults	21.6 (2.8; —)	71.4% female	—	United States	PA^g^ and sleep^g^
Sladek et al [69],^a^ 2020	61 young adults	20.91 (0.36; —)	25% male	51% White or European American, 5% Black or African American, 5% Asian American or Pacific Islander, 10% multiracial, and 6.1% Hispanic or Latinx	United States	Sleep^c,g^
Sperry et al [70],^a^ 2018	49 university students	19.3 (1.7; 18-25)	77.6% female	36.7% White or European American, 8% African American, 8.2% Asian or Pacific Islander, 4.1% biracial, 6.1% preferred not to answer, and 6.1% Hispanic or Latinx	United States	PA^b^
Sperry and Kwapil [71],^a^ 2022	233 young adults	18.81 (1.04; —)	71% female	52% White, 22% Asian, and 13% Black	United States	Sleep^c^
Strahler et al [46],^a,i^ 2016	33 university students	22.8 (3.3; —)	—	—	Germany	PA^c,g^
Titone et al [72],^a^ 2020	107 young adults	21.82 (2.16; 18-27)	55% female	59.8% White, 17.8% Black or African American, 11.2% Asian, 0.9% American Indian or Alaskan Native, 3.7% Biracial, and 3.7% other race	United States	Sleep^g^
von Haaren et al [73],^a^ 2013	29 university students	21.3 (1.7; —)	—	—	Germany	PA^g^
von Haaren et al [74],^a,h^ 2016	61 university students	21.4 (1.8; —)	100% male	—	Germany	PA^g^^,j^
van Woerden and Bruening [47],^a,i^ 2022	805 first-year university students	—	71% female and 29% male	49% White, 11% Black, 14% other, and 27% Hispanic	United States	PA^b^
Walter et al [75],^h^ 2013	23 apprentices	19.43 (1.85; —)	52.2% female and 47.8% male	—	Germany	PA^g^
Wen et al [76],^a^ 2022	96 university students	21.4 (2.92; —)	38.9% male	—	China	PA^g^ and sleep^g^
Wu et al [77],^a^ 2021	1584 university students	—	62% female	—	United States	PA^g^ and sleep^g^
Yap et al [78],^a^ 2022	89 undergraduate students	20.54 (1.64; —)	76.5% female, 20.5% male, and 3% other	84.7% Asian, 9.2% White or European, and 6.1% other race	Australia	Sleep^c,j^

^a^Observational study design.

^b^Data were collected via electronic ecological momentary assessment.

^c^Data were collected via a recall survey (daily, weekly, or longer) or diary.

^d^PA: physical activity.

^e^SB: sedentary behavior.

^f^Not reported.

^g^Data were collected via an accelerometer (or a pedometer).

^h^Randomized controlled trial component within the study design.

^i^Secondary analysis.

^j^Data collection via another method.

^k^Quasi-experimental study design.

### Quality of the Data Set

**Figure 2:**
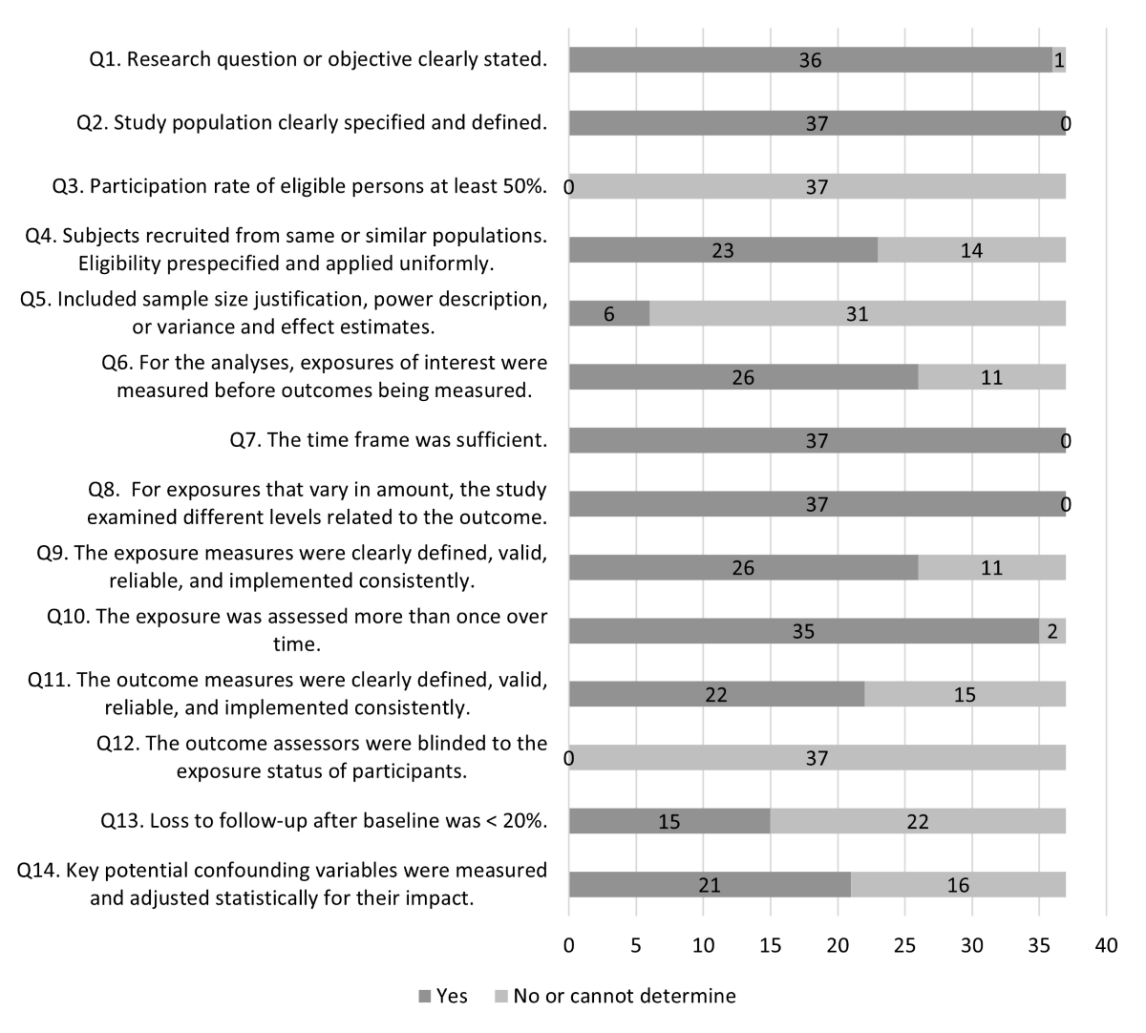
Risk of bias assessment.

The risk of bias assessment resulted in the scoring of 5 reports as good quality [48,50,58,60,78] and 32 reports as fair quality [42-47,49,51-57,59,61-78]. Figure 2 outlines the percentage of articles scoring yes and no or cannot determine for each question from the NHLBI Quality Assessment Tool for Observational Cohort and Cross-Sectional Studies [38]. No study has indicated a participation rate of at least 50%. Most samples were recruited from large populations on college campuses and used a variety of recruitment methods, limiting the ability to track the number of eligible participants. No studies reported blinding of assessors, which was expected, as most studies had observational designs. In total, 6 studies included adequate justification for the selected sample size [50,55,57,58,60,78], such as a power analysis [58,60,78] or another evidence supporting the argument for sample size or number of data points needed for analysis [50,55,57]. A total of 15 studies reported an attrition rate ≤20% [48,50,51,53,55,56,58,60,61,64,67,68,70,74,76]. One of the strengths of eEMA study designs is that the methodology allows for directional analyses of temporality, including for bidirectional relationships; however, only 26 reports clearly delineated that the exposures or predictors were measured before the outcomes were measured [42,45,46,48-50,52,56,58,60-63,65,66,68-78]. Several studies included multiple predictor and outcome variables measured using EMA and other data collection methodologies. Most studies included some psychometric support but lacked complete details for all measures [50,51,56]. Therefore, questions 9 and 11 of the NHLBI quality assessment tool, which ask about the validity and reliability of exposure and outcome measures, were categorized based on overall clarity and psychometric support rather than requiring complete psychometric support described for all measures. Multimedia Appendix 2 [38,39,42-78] provides details on the risk of biases scoring for each article, and Multimedia Appendix 3 [42-78] provides additional details regarding psychometric support.

### Outcomes and Measures

#### Physical Activity

Physical activity was measured in 76% (28/37) of the reports (Table 2). A total of 14 reports included self-report measures delivered via eEMA to assess physical activity [42,43,45,47,48,50,53,55,59,62,65-67,70], and 16 reports included objective measures of physical activity such as accelerometry or pedometry [43,45,46,49,54,56-59,67,68,73-77]. In total, 8 reports included physical activity recall surveys [43,44,46,48,56,59,61,65], with durations varying from daily [51] or weekly recall [44,48] to reporting the average time spent doing physical activity over the past 12 months [46]. Ponnada et al [45] and Sano et al [67] included eEMA and accelerometry measures for physical activity (Table 2).

In 7 reports, the eEMA questions asked participants to select current or recent activities, including physical activity, from a list of activities [42,47,53,55,62,65,70]. Recall time frames included “time of prompt” [47,55,62], “since the last prompt” [70], and “in the last 20 minutes” [65]. In 2 studies, if the current activity was identified as physical activity, then the duration or intensity was also recorded [42,53]. In 5 studies, the duration or intensity of physical activity since the last prompt was recorded [45,48,50,66,67]. A total of 2 studies included measures developed from the select items of previously validated physical activity scales (eg, International Physical Activity Questionnaire and Leisure Time Exercise Questionnaire) [42,48]. Other studies created new items or did not discuss previous psychometric support.

#### Sedentary Behavior

In total, 11% (4/37) of the reports included measures of sedentary behavior, all of which were delivered via eEMA [42,45,48,64]. Of these 4 reports, 2 (50%) included sedentary behavior measured by accelerometry. Specifically, Romanzini et al [64] tested the agreement between sedentary behavior identified by eEMA and accelerometry, and Ponnada et al [45] used accelerometer data to trigger eEMA questions about the duration of sedentary behavior. eEMA measures for sedentary behavior were single-item questions about current activities with sedentary behavior–related options [64] or about the duration of sedentary activity [42,48].

#### Sleep

In total, 43% (16/37) of the reports included measures of sleep. In these 16 reports, sleep was measured using accelerometry (10/16, 63%) [51,52,58,60,67-69,72,76,77], recall diaries or questionnaires (9/16, 56%) [51,52,58,60,61,63,69,71,78], and eEMA (3/16, 19%) [55,62,67]. Most studies included measures of both sleep duration and sleep quality [61,63,71,78]. eEMA questions regarding sleep were newly created items about current activity, in which sleeping was an option [55,62,67]. Yap et al [78] measured sleep via portable electroencephalogram and a self-report daily sleep diary.

#### Multiple Behaviors

Zero studies included measures of physical activity, sedentary behavior, and sleep. In total, 3 studies included measures of physical activity and sedentary behavior [42,45,48]. All 3 of these studies included eEMA to collect data on physical activity and sedentary behavior. In addition, 8 studies included measures of physical activity and sleep [55,58,61,62,67,68,76,77]. Of these studies, 3 used eEMA [55,62,67], 5 used accelerometry [58,67,68,76,77], and 1 used recall surveys [61] to measure physical activity and sleep. Of the studies that included 2 movement behaviors, 10 (91%) of 11 studies used the same data collection modalities (eg, eEMA, accelerometry, and recall surveys) to measure both of the included movement behaviors [42,45,48,55,61,62,67,68,76,77], and 4 studies included >1 data collection modality, such as eEMA with accelerometry [45,48,58,67].

#### Other Outcomes

eEMA was often used to measure the correlates of movement behaviors rather than or in addition to the movement behaviors themselves. The most common correlates measured using eEMA were related to mood, affect, and feelings such as stress [57,58,61,66-69,74,77,78]; negative and positive affective states [49,51,52,69,71,73,75,76]; urges [44,51]; depressive symptoms [54,68,72,76]; anxious states [50,54,68,76]; fatigue [46,54]; and other emotional states [50,55,63,67,72,77], which allows not only the analysis of these variables with movement behaviors but also the exploration of emotional inertia, variability, and stability [50,76]. eEMA was also used to measure reflective and reactive cognitive processes specific to physical activity, sedentary behavior, or sleep [45,49,53,56,60]. eEMA items were commonly used to collect data on current activity or behavior [42,47,49,55,62,64,65,67,77], location or environment [43,47,49,59,64,77], social context or interactions [43,47,49,59,64,65,67,70], additional self-regulatory cognitive processes [49,63,71], and other behaviors such as dietary behaviors [42,47,57,67-70] and smoking or nicotine intake [44,70]. Multimedia Appendix 3 provides the additional outcomes measured using eEMA.

### Analytical Approaches

Measures of the movement behaviors were considered primary outcomes in 13 analyses [42,43,47,48,52,56,58-60,64,66,71,78]. Most of these analyses (11/13, 85%) considered the temporality in relationships between predictors and repeated outcome measures using methods to account for clustering and longitudinal study design, including mixed effect models, multilevel models, or generalized estimating equations [42,43,47,52,56,58-60,66,71,78]. Moreover, Sperry et al [71] leveraged a novel dynamic structural equation modeling approach to examine within-individual and cross-lagged relations. Of the 11 studies in which measures of 2 movement behaviors were included [42,45,48,55,58,61,62,67,68,76,77], only 3 (N=11, 27%) studies integrated both movement behaviors into a single analysis [42,61,68]. In 23 analyses, measures of the movement behaviors were considered as secondary outcomes or independent predictor variables [44,46,49-51,53-55,57,61-63,65,67-70,72-77]. Most of these studies (18/23, 78%) also considered temporality using similar methods [44,46,50,53-55,57,61-63,65,68-70,72,74-76].

### eEMA Procedures

Participant incentives were noted in 68% (25/37) of the reports (Table 3). Among these reports, the incentives were in the form of monetary compensation such as gift cards or cash (12/25, 48%) [42,43,45,47,49,52,55,59,61,63,67,76], course credit (5/25, 20%) [50,53,70,74,77], or a combination of both (8/25, 32%) [46,48,51,56,60,65,66,71]. In 64% (16/25) of the studies with incentives, the incentive amounts were dependent on a minimum completion threshold or prorated for completing questions and returning equipment [42,43,45,49-53,56,59,60,63,65,67,71,74]. For example, in the study by Bedard et al [49], participants received a US $10 Starbucks gift card for completion of the initial questionnaire and for agreeing to wear an accelerometer. They also received US $1 for each prompt completion, with a maximum of US $5 per day, in the form of a Starbucks gift card. Bernstein et al [50] awarded academic credits that included full credit for at least 75% completion of prompts, with prorated amounts for completion rates of <75%. Other researchers used raffles where the entries were dependent on the completion rates of questionnaires or prompts [43,59,60].

**Table 3:** Electronic ecological momentary assessment procedures.

Study	Incentives	Technology	Prompt modality	Monitoring period	Prompt design	Response window and reminders
Andorko et al [48]	Course extra credit and monetary compensation	Smartphone^a,b^; SurveySignal (SurveySignal) platform	Text message with link	1 week; 7:30 AM-10:30 PM	Time based (RIC^c^); 6 per day; 2-hour intervals	20 minutes
Bedard et al [49]	US $10 gift card for baseline questionnaire and to wear accelerometer; US $1 gift card per prompt^d^	Smartphone^a,b^^;^ ilumivu (ilumivu) app	Smartphone notification	5 days; 9 AM-11 PM	Time based (RIC); 7 per day; 2-hour intervals	—^e^
Bernstein et al [50]	Course credit^d^	Smartphone^a^; LifeData (LifeData, LLC) app	Smartphone notification	5 days per wave; 3 waves 4 weeks apart	Time based; 5 per day	—
Bruening et al [42]	Up to US $80^d^	Smartphone^a,b,f^; devilSPARC app [42]; Twilio (Twilio Inc) web service	Text message with link	4 days (Wednesday-Saturday); 9 AM-10 PM	Time based (RIC); 8 per day^g^; 3- to 4-hour intervals	35 minutes; 2 reminders
Burke et al [51]	US $15 or course credit^d^	Smartphone^a,b^	Text message with link	10 days; custom 12-hour window	Time based (RIC); 9 per day; 4-hour intervals; at least 1.5 hours apart	Incentive to respond in 30 minutes
Das-Friebel et al [52]	£5 (US $6.34) for baseline questionnaire; £2.50 (US $3.17) per day^d^	Smartphone^a^; ilumivu (ilumivu) app	Smartphone notification	14 days; 8 AM-10 PM weekdays and 10 AM-10 PM weekends	Time based, 6 random times per day^g^; at least 1 hour apart	20 minutes
Gilchrist et al [53]	Course credit and extra credit^d^	Smartphone^a,b^; PACO (Personal Analytics Companion Mobile; PACO Developers) app	Smartphone notification	7 days; custom 12-hour window	Time based; 7 random times per day; at least 1 hour apart	45 minutes
Kim et al [54]	—	Wrist-worn computer^f^	Beep on watch	2 days	Time based (RIC) and event based; every 2 hours within 12 minutes (± 12 minutes)	20 minutes
Kono et al [55]	3000 yen (US $21.47)	Smartphone^a,b^; LifeData (LifeData, LLC) app	Smartphone notification	7 days; 8 AM-10 PM	Time based; 7 per day; 2-hour intervals	30 minutes
MacIntyre et al [56]	Course credit and up to US $20^d^	Smartphone^a,b,f^; LifeData (LifeData, LLC) app	Smartphone notification	7 days; 9 AM-9 PM	Time based (RIC) and event based, 5 per day^g^; at least 2 hours apart	60 minutes
Maher et al [57]	—	Smartphone^b,f^; MovisensXS (movisens GmbH) app	Smartphone notification	7 days; 9:30 AM-10:30 PM	Time based (RIC); 5 per day; 1-hour intervals	15 minutes; 3 reminders
Maher et al [58]	Same procedures as Maher et al [57]	Same procedures as Maher et al [57]	Same procedures as Maher et al [57]	Same procedures as Maher et al [57]	Same procedures as Maher et al [57]	Same procedures as Maher et al [57]
Marquet et al [43]	Entrance in a raffle of 8 gift cards worth US $50^d^	Smartphone; Pacer (Pacer Health Inc) step counting app; PACO (Personal Analytics Companion Mobile; PACO Developers) app	Smartphone notification	7 days	Time based; 3 per day	60 minutes
Marquet et al [59]	Same procedures as Marquet et al [43]	Same procedures as Marquet et al [43]	Same procedures as Marquet et al [43]	Same procedures as Marquet et al [43]	Same procedures as Marquet et al [43]	Same procedures as Marquet et al [43]
Mead, and Irish [60]	Course credit and entry to raffle for 2 US $50 cash prizes^d^	Mobile phone^a,b^	Text message with link	7 days	Time based and event based; 4 per day; customized times	None; email reminders
Miller et al [61]	US $120	Handheld computer^b,f^	Computer alarm	13 days (before, during, and after vaccination)	Time based; 4 per day; custom wake time	60 minutes
Milyavskaya et al [62]; study 1 only	—	Smartphone^a,b^	Text message with link	7 days; 10 AM-10 PM	Time based (RIC); 5 per day	—
Nadell et al [44]	—	Handheld computer^b,f^	Computer notification	7 days	Time based and event based; 5-7 random times per day	—
Parsons et al [63]	Monetary vouchers plus additional compensation^d^	Smartphone^a^; Metricwire (Metricwire Inc) app^b^	Smartphone notification	7 days; 10 AM-10 PM	Time based (RIC); 4 per day; initial between 10 AM and 12 PM; at least 2 hours apart	20 minutes
Ponnada et al [45]	US $20 per month for wearing smartwatch; up to US $80 and was able to keep the smartwatch^d^	Smartwatch^f^; smartphone^a^; Study designed app [45]^b^	Smartphone and smartwatch notifications	1 month of microassessments including two 4-day burst periods with traditional momentary assessments; custom waketime window	Time based and event based microassessments: 4 random times per hour at least 8 minutes apart; Burst periods: 1 per hour	20 seconds
Romanzini et al [64]	—	Smartphone^a^; ilumivu (ilumivu) app	Smartphone notification	7 days	Time based; weekdays: 8 per day; weekends: 9 per day; random every 2 hours	None; 3 reminders
Runyan et al [65]	Course credit; US $5 gift card for device return and compliance^d^	Smartphone; iPod Touch (Apple Inc)^a,b^ iHabit [65] app	Smartphone notification	1 week per wave; 3 waves throughout an academic semester; 6 AM-11 PM	Time based; 5-7 per day	—
Sala et al [66]	Course credit or payment	Phone^a,b^; automated telephone system TelEMA [66]	Automated telephone calls	7 days; custom 12-hour window	Time based (RIC); 4 per day; 3-hour intervals	1.5 hours; voicemail reminder
Sano et al [67]	Monetary compensation^d^	Web-enabled device^a^	Email with link	1 month	Event based; 2 per day	12 hours; email reminder
Shah et al [68]	—	Smartphone^a^; *BrainE* (University of California, San Diego, Business Affairs) app	Smartphone notification	30 days; 8 AM-8 PM	Time based; 4 per day; every 4 hours	—
Sladek et al [69]	—	Web-enabled smartphone^a^	Unspecified	8 days	Time based and event based; 4-5 per day	—
Sperry et al [70]	Course credit	Tablet^b,^^f^	Tablet flashing and alarm	1 day; 6-9 hours	Time based; 12 random times approximately every 45 minutes	—
Sperry, and Kwapil [71]	Course credit. Raffle for US $100 gift card^d^	Smartphone^a,b^	Smartphone notification	14 days; 10 AM-10 PM	Time based (RIC), 8 per day; 1.5-hour intervals	10 minutes
Strahler et al [46]	Course credit or 50 euros	iPod touch^b,^^f^	iPod notification	5 days per wave, 2 waves including 1 at the beginning of the academic semester and 1 during final examination preparation	Time based; 5 per day; every 3-4 hours	—
Titone et al [72]	—	Mobile phone^a,b^	Text message	20 days including 5 at baseline, 10 during the intervention, and 5 postintervention	Time-based varying intervals 3 per day (morning, afternoon, and evening)	—
von Haaren et al [73]	—	PDA^f^; My Experience (movisens GmbH)	Device alarm (vibrating)	2 days; 10 AM-10 PM	Time based; every 2 hours	15 minutes; 2 reminders
von Haaren et al [74]	Course credit^d^	Smartphone^f^; My Experience (movisens GmbH)	Smartphone notification	2 days per wave; 3 waves (1 preintervention and 2 postintervention during examination weeks);10 AM-10 PM	Time based; every 2 hours within 5 minutes	None; 3 reminders
van Woerden, and Bruening [47]	Gift cards and product incentives for the larger study	Smartphone^a,f^; devilSPARC [47] app	Smartphone notification	4 days (3 weekdays and 1 weekend day) per wave; 4 waves (September, October, February, and March)	Time based (RIC); 8 per day; 3- to 4-hour intervals; at least 30 minutes apart	40 minutes
Walter et al [75]	—	PDA^f^; My Experience (movisens GmbH)	Device audible signal or vibration	3 days (weekdays) per wave; 5 waves (preintervention; weeks 2, 6, and 10; and postintervention)	Time based and event based; 5 per day (within 15 minutes of waking, 10 AM, 1 PM, 4 PM, and 8 PM) and immediately, 20 minutes, and 40 minutes after training sessions	20 minutes; 3 reminders
Wen et al [76]	1-2 yuan (US $0.14-$0.28) per questionnaire	Not specified^b^; WeChat (Tencent) app	App notification of message with link	7 days; 9 AM-8 PM	Time based; 5 times per day (9 AM, 11 AM, 3 PM, 5 PM, and 8 PM)	60 minutes
Wu et al [77]	Course credit	Smartphone^a^; Beiwe (Harvard University Onnela Lab) app, email, or eDiary	Smartphone notification or email	3 weeks; 9 AM-9 PM	Time based; 5 times per day^g^ (9 AM, 12 PM, 3 PM, 6 PM, and 9 PM)	—
Yap et al [78]	—	Not specified^b^; Metricwire (Metricwire Inc) app	App notification. backup SMS text messaging, or phone call	15 days	Time based; 4 per day; (morning, afternoon, evening, and bedtime)	90 seconds; hourly reminders

^a^Device provided by the participant.

^b^Training provided to the participant.

^c^RIC: random interval contingent.

^d^Incentive prorated or dependent on minimum threshold compliance.

^e^Not reported.

^f^Device provided by the study.

^g^Ecological momentary assessment prompts included a daily recall survey.

The devices used for eEMA delivery varied, with most studies using mobile phones or smartphones (24/35, 69%) often provided by the participant (Table 3). The study provided devices, including smartwatches (2/35, 6%) [45,54], smartphones (6/35, 17%) [42,47,56-58,74], and handheld computers or tablets (6/35, 17%) [44,46,61,70,73,75]. Other researchers used web-based questionnaires available via any web-enabled device [67,77] or via an automated telephone system that provided prompts and question delivery [66]. Frequently used mobile apps included eEMA apps by Ilumivu Inc (3/35, 9%) [49,52,64], movisens GmbH (5/35, 14%) [57,58,73-75], LifeData, LLC (3/35, 9%) [50,55,56], and Metricwire Inc (2/35, 6%) [63,78] as well as the devilSPARC app (2/35, 6%) [42,47] and PACO the Personal Analytics Companion app (3/35, 9%) [43,53,59]. Almost two-thirds of the reports (23/37, 62%) included some form of participant training, such as assistance with downloading the mobile app or instructions on using the device or app to answer the prompts [42,44-46,48,49,51,53,55-58,60-63,65,66,70-72,76,78].

Prompting methodology was largely dependent on the selected device. Most studies included prompting via a smartphone or app notification with eEMA items (21/37, 57%) [43,45,47,49,50,52,53,55-59,63-65,68,71,74,76-78], text messages with links to surveys (7/37, 19%) [42,48,51,60,62,72,78], or emails with links to surveys (2/37, 5%) [67,77]. Services such as Survey Signal [48] and Twilio were used to send messages [42] with links to the surveys. Prompting was also conducted through phone calls (2/37, 5%) [66,78] and other notifications on devices such as computers, tablets, or watches (8/37, 22%) [44-46,54,61,70,73,75]. In total, 4 reports described the use of multiple prompting modalities to allow for participant preference, reminders, or backup in the case of technological failures [45,60,77,78]. For example, Yap et al [78] included app notifications with messaging and phone calls as backup methods.

EMA data were collected for periods ranging from 1 day to 1 month, with a mean average of 11.3 (SD 7.8) days of monitoring (Table 3). A majority of the study procedures (27/35, 77%) used only time-based prompting schedules either with researcher-scheduled prompts or random interval contingent prompting, which often incorporated a set minimum amount of time between prompts (eg, 45 minutes; Table 3). A total of 7 studies used a combination of time-based and event-based prompting schedules [44,45,54,56,60,64,75], and 1 study used only event-based prompting [67]. In these studies, triggering events were defined as waketime and bedtime, except for Nadell et al [44], who used smoking as the event trigger, and Walter et al [75] defined the physical activity intervention training sessions as the event trigger. In studies using time-based prompts, participants were sent 2 to 12 prompts per wake period for the duration of the study. In contrast, Ponnada et al [45] sent participants up to 4 prompts per hour on *burst days*. All prompts were restricted to the wake period, which was defined by either the participant or researcher ahead of time.

Specific design features to reduce bias and participant burden of the EMA protocol included strategies such as limiting the amount of time participants had to respond before the prompt expired. The time to respond window ranged from 20 seconds [45] to 90 minutes [66]. The authors also reported allowing participants to postpone the prompt twice for 10 minutes [54], accepting responses to prompts up to 60 minutes before or after the scheduled delivery time [61], allowing participants to *undo* their selection for 3 seconds [45], and sending participant feedback about response rates to encourage participation [55]. The anticipated time to complete the questions varied. Some questions were designed to be answered in 2 to 3 minutes [68,76], whereas others were designed to be answered with a single selection (eg, *Yes*, *No*, or *Sort of*), thus requiring minimal time to complete [45]. Customizable features were used to reduce participant burden, such as customizable wake time windows [45,51,53,66] and options for reminder modality [66], prompt modality [77], and type of device [65,67,77] including phones, tablets, or computers. Table 3 provides additional information regarding eEMA procedures for the included studies (Table 3).

### Compliance

Ideal EMA reporting includes factors related to the participant’s ability to comply with EMA prompts and any barriers that they may experience. Reporting rates related to attrition, prompts delivered, latency, response rates, and missing data provided insight into overall eEMA compliance (Multimedia Appendix 3). Of the 37 reports reviewed, 5 (14%) indicated participant attrition across days or waves of monitoring [47,65,67,75,77]. In total, 2 reports included prompt delivery data to indicate the number or rate of prompts delivered to participants [45,58]. Latency rates, which provide insight into the time between prompt delivery and response, were presented in 14% (5/37) of the reviewed articles [42,45,47,53,55]; however, 60% (22/37) of the articles included response rates. Among reports that included an overall response rate, these rates ranged from 54.4% [55] to 94.7% [69]. Rather than the overall response rate, some authors have reported the response rate or average number of prompts answered per participant [47,53,58,59,66,68]. There was a substantial decrease in participation over time among studies that reported attrition or compliance. For example, Walter et al [75] reported that the response rates decreased from 72% at baseline to 38% during the final wave. Similarly, Runyan et al [65] reported a 40.1% response rate during week 1 and 19.1% during week 3. Some study procedures included strategies to increase compliance, such as email reminders of incentives if response rates dropped below a threshold [63], daily reminders for incomplete surveys [60], and reminders following prompts when surveys were not completed (10/35, 29%) [42,57,58,60,64,66,67,73-75,78].

Finally, discussions of missing data were present in 76% (28/37) of articles. Approximately one-third (12/37, 32%) of the articles excluded participants from the analysis based on low compliance. The minimum compliance thresholds ranged from 20% [56] to 80% [43]. In total, (7/37, 19% of the articles included brief descriptions of additional techniques to address missing data [46,56,59,63,68,71,72]. Moreover, 30% (11/37) of the articles provided reasoning for low compliance or examination of specific patterns of missing data [44,47,52,53,57,58,62,64,65,73,75]. For example, Van Woerden et al [47] examined compliance based on demographic characteristics and prompting protocol. They found lower compliance before midday, on weekends, and after the initial wave of data collection [47]. Walter et al [75] found low compliance immediately before and after an in-person physical activity intervention delivered by the researchers. Maher et al [57] identified missing data related to gaps in the prompting protocol and no significant compliance variations based on time of day, weekday or weekend days, steps taken, sex, or BMI. The authors also pointed out that participants were instructed to ignore prompts during incompatible activities such as driving or taking an academic examination [52,53,64].

## Discussion

### Principal Findings

We examined how the eEMA methodology was used in the study of physical activity, sedentary behavior, and sleep in young adults. The findings of this review can guide the precise and practical implementation of the eEMA methodology in future research. Our review resulted in 37 reports of 35 original studies. We found that eEMA has been used in this area with increasing frequency in recent years, which is likely a reflection of the increasing availability and usability of eEMA-compatible technologies. This increase was most evident in sleep research, in which 13 (81%) of 16 reports were published since 2019. In 2016, a review of EMA physical activity and diet research conducted with young people showed that only half of the devices used in eEMA research were mobile phones, whereas the other half used handheld computers [40]. In this review, 80% (28/35) of the studies reported the use of smartphones or smartphone-compatible technology (eg, mobile apps or web-based platforms), and 20% (7/35) of the studies reported using other devices such as handheld or wrist-worn computers or smartwatches (Table 3). This shift reflects the impact of evolving technological innovations on research methodologies, further highlighting the importance of regular methodological reviews in synthesizing current practices in a rapidly evolving field.

In this review, there were more reports of research on physical activity (28/37, 76%) than on sedentary behavior (4/37, 11%) or sleep (16/37, 43%). We can only speculate that this may be because physical activity has a longer history in health behavioral epidemiology and application within health psychology than sleep research and sedentary behavior research. In addition, eEMA is well suited for the momentary assessment of physical activity behaviors and correlates. In contrast, objective measures may be more practical for assessing sleep duration. At the same time, many dimensions of sleep quality and correlates of sleep are easily assessed using eEMA. In this review, physical activity and sleep were measured more frequently using objective (eg, accelerometer) data collection methods than self-report via eEMA. A majority of study reports (23/37, 62%) included an objective measure of at least 1 movement behavior, whereas 38% (14/37) relied solely on subjective measures.

eEMA was most frequently used to measure constructs correlated with physical activity, sedentary behavior, or sleep such as psychological, social, and contextual factors, rather than the movement behavior itself. This was consistent with previous systematic reviews in which common constructs measured via EMA included cognitive processes and affect or mood [41]. Given the psychological vulnerability during young adulthood, the examination of such momentary psychological constructs in real time and in relation to movement behaviors is particularly important for this population. Furthermore, psychological, social, and contextual constructs lend themselves well to data collection via eEMA because of their subjective nature and natural intraindividual variability. EMA methodologies allow the exploration of dynamic modeling [31] to better represent how factors such as affect [79,80], identity [81], intention [82], and social or environmental context interact with movement behaviors [83].

Most studies (32/37, 86%) were of fair quality and used longitudinal observational designs. Although the NHLBI Quality Assessment Tool for Observational Cohort and Cross-Sectional Studies [38] was selected for its reliability and incorporation of temporality, attrition, and confounding variables, it proved challenging to use with some of the unique features of eEMA studies and the relative infancy of the field. For example, questions 9 and 11 from the quality assessment tool asked whether the exposure and outcome “measures were clearly defined, valid, reliable, and implemented consistently” [38]. The eEMA studies in this review included several data collection methodologies such as full-length self-report questionnaires, eEMA measures, and objective measures to assess numerous predictor and outcome variables, sometimes including bidirectional relationships. As such, categorizing psychometric support into yes or no and cannot determine proved to be less practical. As eEMA is a developing field, there are few reliable and validated measures specifically tested for eEMA delivery. Psychometric support for EMA measures or psychometric findings was reported in 35% (13/37) of the articles [49,52,53,55,62-64,66,71,73,75,76,78]. Almost half of the studies (17/37, 46%) did not discuss psychometric support for the eEMA items or included newly created items without reporting psychometric findings. Others have selected items from reliable and well-validated scales typically presented in a longer recall format. Multimedia Appendix 3 presents additional information on psychometric support. Our findings align with those of Degroote et al [34], who in 2020 documented a lack of psychometric support for eEMA measures used in physical activity and sedentary behavior research. As the use of eEMA increases, it is important to continue to develop a body of evidence regarding the measures used in eEMA by evaluating and reporting psychometrics to ensure that the items and scales used to assess physical activity, sedentary behavior, and sleep constructs are reliable and valid.

We also found substantial variations in other areas of reporting on eEMA procedures. Although most reports included some description of participant incentives, technology used, prompt modality, monitoring period, and prompting design (including frequency), the depth of the descriptions varied greatly. Other areas of reporting were inconsistent, suggesting a continued lack of standardized definitions and reporting for eEMA studies in this area. For example, we found low rates (<15%) of reporting attrition, prompts delivery rates, and latency time. Owing to the longitudinal nature and frequent measures involved in eEMA studies, descriptions of such procedures and results provide an important context needed to interpret findings and determine the risk of bias. For example, simple pre-post calculations of attrition lacked sufficient detail to paint a larger picture of participation throughout the study. In 2016, Liao et al [40] recognized the lack of standardized reporting and developed the Checklist for Reporting EMA Studies. The strength of this checklist lies in the standardized definitions and unique categories specific for EMA studies to guide researchers in the collection of important data metrics and the reporting of EMA studies. As such, this checklist provided a framework for data collection and synthesis in this review.

The results of our systematic review also highlight important methodological considerations for future studies using eEMA to assess physical activity, sedentary behavior, or sleep among young adults. Studies using eEMA with short time-based intervals over extended periods produced data with high temporal resolution. For example, Ponnada et al [45] studied 81 participants for 6 months that involved the delivery of 662,397 EMA questions. Using simple statistics, such as calculating mean responses per day or per study period, may diminish the unique benefits of the eEMA methodology, whereas more advanced methods, such as mixed effect models, can be used to assess temporality when studies are designed to have repeated observations. Repeated data require more extensive protocols for data cleaning and handling of missing data because response rates can be impacted by the prompting schedule [40], among other factors. For example, different schedule constraints, such as those because of college courses or working a third shift, can affect a participant’s ability to respond to some prompting schedules and result in more missingness at certain times of the day. Future studies should address the unique schedules of study participants through customizable features, set quiet times, or additional pilot testing to determine participant needs. Moreover, it is important for studies to examine the risk of different missingness patterns (eg, missing not at random, missing at random, and missing completely at random). The results from analyses that ignore nonrandom missingness may not be generalizable to the entire study population [40]. Thus, the analysis and reporting of missing data are of particular importance in EMA methodology, as it can highlight demographic or time-varying variables where missing patterns might be discerned. To leverage the full potential of these fine-scale longitudinal data, particularly given the complexity of collecting real-time data, it is necessary to build a research team with diverse expertise that includes expertise in data management and advanced longitudinal data analysis.

Another important consideration in the collection of complex real-time movement data is the integration of multiple data collection methodologies such as accelerometry and eEMA. Several studies have demonstrated how this approach can be used to refine eEMA questions for contextual relevance [45] or to validate movement behavior data [64,67]. In a recent study with adults, eEMA and actigraphy were used to explore the associations of affect with movement behavior compositions and reallocations within the 24-hour period. The associations between affect and 24-hour movement behavior composition varied by activity and behavior reallocation patterns, suggesting complex interrelationships among the behaviors within 24-hour compositions [84]. The development of eEMA technologies and their potential integration with accelerometry have enabled the study of movement behaviors and their correlates within a 24-hour context. Although the focus on movement behaviors within the 24-hour period has increased among youth and adult populations, no studies in this review of eEMA with young adults have measured all 3 movement behaviors. This is likely because 24-hour movement behaviors, clustered together, are a relatively recent development, tracing back to work in the mid-2010s and only receiving policy attention in 2016 first with the Canadian Child and Youth Guidelines [26]. As it is relatively novel in conceptualization, there are relatively few EMA studies exploring the composition of movement behaviors within a 24-hour period. Thus, there remains a critical need and great opportunity to use eEMA methodologies to explore the complex interconnectedness and potential increased effects of 24-hour movement behaviors on health and well-being in young adults.

### Implementation

Data collection specific to the implementation of eEMA can provide valuable insights to support participant engagement. Attrition and compliance issues can pose serious problems for research, including concerns regarding the reliability and validity of the research findings. The reasons for attrition and poor compliance may be related to acceptability and usability issues with the procedures or technology. Young adults, as part of Generation Z, have experienced their entire childhood in the digital age and are frequently early adopters of technology [85]. With an affinity toward technology, it is often assumed that young adult participants will experience an easy uptake of research that integrates technology. However, usability and acceptability are precursors to compliance. As highlighted by Liao et al [40], clear descriptions of attrition, compliance, and missingness, including a thorough examination of explanations, are necessary to interpret the results of EMA studies.

Among the studies reviewed, anecdotal reasons for low compliance were often presented in the form of researcher comments to provide insight into user experience; however, 3 studies sought to quantify the participant experience more concretely through poststudy interviews or surveys [45,49,65]. For example, Bedard et al [49] conducted a voluntary, anonymous web-based process evaluation survey to gather data on the acceptability and receptivity from participants’ perspectives. The length of the study and the number of prompts received were rated as appropriate, but some respondents (16/47, 34%) thought there were “somewhat” or “far too many” prompts. Ponnada et al [45] conducted feasibility testing using semistructured interviews, allowing the team to modify the prompt availability time and device notification features before the implementation of their main study. Such feasibility testing is critical for the successful implementation of eEMA methodologies and can contribute to the understanding of attrition, compliance, and missingness.

In addition, the reporting of acceptability and feasibility contributes to the development of methodological knowledge regarding the implementation of eEMA in young adults. In this review, several strategies to reduce participant burden and increase compliance were identified, such as using sensory data to trigger only relevant EMA questions [45,86], allowing the customization of “wake” periods, and providing features to skip or postpone responses. These strategies are consistent with findings from digital health research on young adults, which has demonstrated that young adults prefer research schedules that fit within their daily schedule and avoid irrelevant and unnecessarily redundant content [45,87].

As evidence regarding the use of eEMA in physical activity, sedentary behavior, and sleep research in young adults develops, it is important to consider the populations included in the existing studies. Most samples in this review were college based, which inherently may limit the generalizability of the findings, as young adults who directly enter the workforce after high school are underrepresented. Young adults in college often have set class schedules and access to campus recreational facilities, intramural and extramural sports opportunities, and a variety of clubs involving physical activity (eg, hiking and frisbees). Health behavior opportunities and resources differ for young adults who directly enter the workforce. Furthermore, the studies in this review often included limited descriptions of the sample. Only 1 study [78] included sex or gender information beyond a dichotomous perspective of male or men participants and female or women participants, and only 62% (23/37) of the reports included racial or ethnicity demographics of the sample. The most frequently reported racial demographics were White or European American. This occurred despite this demographic not being the largest in the world [88], reflecting an overrepresentation of this group in such research. This is because most studies were conducted in Northern American and European countries. Given the limited demographic and geographic diversity among the studies presented and the continuing digital divide worldwide in which half of the world’s population lacks adequate internet access [89], future studies need to be purposeful in the recruitment and inclusion of diverse samples to increase the generalizability of evidence in this area to young adults across the globe.

### Limitations

Despite the thorough systematic search and review process, some relevant studies may have been missed. For this review, eEMA was defined as including prompts sent more than once per day on an electronic device. This definition was used to emphasize studies examining momentary experiences rather than allowing for larger recall durations and diary methodologies; however, this may have limited the research on sleep, as self-reported sleep is often measured using a daily morning recall. We adhered to a strict age range for the young adult life stage based on the Society for Adolescent Health Medicine’s definition of young adult [11]; therefore, studies with slightly older or younger samples were excluded [84,90]. The lack of standardized definitions, specific quality coding metrics, and reporting for EMA studies may have resulted in the exclusion of interesting studies and an increase in reporting bias, thereby affecting the risk of bias and confidence in this synthesis. The inconsistency of evidence because of methodological variations introduced substantial heterogeneity in this review and speaks to the diverse uses of eEMA in movement behavior research. Finally, the generalizability of the findings should take the limited demographic and geographic diversity in the reviewed studies into account.

### Conclusions

In this systematic review, we described the eEMA methodology used in physical activity, sedentary behavior, and sleep research in young adults. Recent technological advances have made eEMA an increasingly useful methodology for collecting data regarding movement behaviors and their correlates, particularly among young adults. As eEMA research increases, standardized and thorough reporting of features unique to eEMA are critical to ensure accurate interpretation of findings by the reader. As movement behavior research shifts toward acknowledging the interconnectedness of behaviors within the 24-hour period, eEMA research in this area with young adults is needed. Existing studies lacked demographic and geographical diversity, suggesting an underuse of technological potential to reach other populations. The findings from this systematic review inform the design, implementation, and reporting of the eEMA methodology in physical activity, sedentary behavior, and sleep research in young adults.

## Appendix

Multimedia Appendix 1 Full search strategy.

Multimedia Appendix 2 Risk of bias assessment.

Multimedia Appendix 3 Outcomes, psychometric support, and compliance.

## Abbreviations

eEMA: electronic ecological momentary assessment
EMA: ecological momentary assessment
NHLBI: National Heart, Lung, and Blood Institute
PRISMA: Preferred Reporting Items for Systematic Reviews and Meta-Analyses

## Data Availability

The data generated or analyzed during this study are included in the published article and appendices.
