# Supplementary material for: Use of Electronic Ecological Momentary Assessment Methodologies in Physical Activity, Sedentary Behavior, and Sleep Research in Young Adults: Systematic Review
Source: J Med Internet Res. 2023 Jun 29;25:e46783. doi: 10.2196/46783 (PMC10365632; doi:10.2196/46783)
Supplement: Multimedia Appendix 1 [file jmir_v25i1e46783_app1.docx]

# **Appendix 1.** Full Search Strategy

Original Search – Conducted August 11 (2021)

Update Search – Conducted August 25 (2022)

## PubMed/MEDLINE

(“Exercise”[Mesh] OR “physical activity” OR exercise OR Walking[Mesh] OR walking OR Running[Mesh] OR running OR Bicycling[Mesh] OR bicycling OR cycling OR “active transport” OR Sleep[Mesh] OR sleep OR sleeping OR “Sedentary Behavior”[Mesh] OR “sedentary behavior” OR sedentary OR sitting OR “physical inactivity” OR “Screen Time”[Mesh] OR “screen time” OR “Video Games”[Mesh] OR “video games” OR videogames OR “video gaming” OR video gaming OR video-games OR video-gaming OR gaming)

AND

(“Ecological Momentary Assessment”[Mesh] OR “ecological momentary assessment” OR “ecological momentary assessments” OR EMA OR mEMA OR “electronic diary” OR “electronic diaries” OR “ambulatory assessment” OR “ambulatory monitoring” OR “momentary assessment” OR “experience sampling method”)

AND

(“Young Adult”[Mesh] OR “young adult” OR “young adults” OR “emerging adult” OR “emerging adults” OR “new adult” OR “new adults” OR “college student” OR “college students” OR undergraduate* OR “university student” OR “university students” OR “young adulthood” OR “new adulthood” OR “emerging adulthood”)

## PsycINFO

(DE “Exercise” OR exercise OR DE “Physical Activity” OR “physical activity” OR DE “Walking” OR walking OR DE “Running” OR running OR cycling OR bicycling OR “active transport” OR DE “Sedentary Behavior” OR “sedentary behavior” OR sedentary OR “physical inactivity” OR DE “Sleep” OR sleep OR sleeping OR DE “screen time” OR “screen time” OR DE “Computer Games” OR “video games” OR videogames OR video-games OR “video gaming” OR videogaming OR video-gaming OR gaming)

AND

(DE “Ecological Momentary Assessment” OR “ecological momentary assessment” OR “ecological momentary assessments” OR EMA OR mEMA OR “electronic diary” OR “electronic diaries” OR “ambulatory assessment” OR “ambulatory monitoring” OR “momentary assessment” OR “experience sampling method”)

AND

(DE “Emerging Adulthood” OR “young adult” OR “young adults” OR “emerging adult” OR “emerging adults” OR “new adult” OR “new adults” OR “college student” OR “college students” OR undergraduate* OR “university student” OR “university students” OR “young adulthood” OR “new adulthood” OR “emerging adulthood”)

## EMBASE

(‘Exercise’/exp OR exercise OR ‘Physical Activity’/exp OR ‘physical activity’ OR ‘Walking’/exp OR walking OR ‘Running’/exp OR running OR ‘Cycling’/exp OR cycling OR bicycling OR ‘active transport’ OR ‘Sedentary Lifestyle’/exp OR ‘sedentary behavior’ or sedentary OR ‘Physical Inactivity’/exp OR ‘physical inactivity’ or ‘Sleep’/exp OR sleep OR sleeping OR ‘Screen Time’/exp OR ‘screen time’ OR ‘Video Game’/exp OR ‘video games’ OR videogames OR video-games OR ‘video gaming’ OR videogaming OR video-gaming OR gaming)

AND

(‘Ecological Momentary Assessment’/exp OR ‘ecological momentary assessment’ OR ‘ecological momentary assessments’ OR EMA OR mEMA OR ‘electronic diary’ OR ‘electronic diaries’ OR ‘ambulatory assessment’ OR ‘ambulatory monitoring’ OR ‘momentary assessment’ OR ‘experience sampling method’)

AND

(‘Young Adult’/exp OR “young adult” OR “young adults” OR “emerging adult” OR “emerging adults” OR “new adult” OR “new adults” OR ‘College Student’/exp OR “college student” OR “college students” OR ‘Undergraduate Student’/exp OR undergraduate* OR ‘University Student’/exp OR “university student” OR “university students” OR “young adulthood” OR “new adulthood” OR “emerging adulthood”)

## Web of Science

(“physical activity” OR exercise OR walking OR running OR bicycling OR cycling OR “active transport” OR sleep OR sleeping OR “sedentary behavior” OR sedentary OR sitting OR “physical inactivity” OR “screen time” OR “video games” OR videogames OR “video gaming” OR video gaming OR video-games OR video-gaming OR gaming)

AND

(“ecological momentary assessment” OR “ecological momentary assessments” OR EMA OR mEMA OR “electronic diary” OR “electronic diaries” OR “ambulatory assessment” OR “ambulatory monitoring” OR “momentary assessment” OR “experience sampling method”)

AND

(“young adult” OR “young adults” OR “emerging adult” OR “emerging adults” OR “new adult” OR “new adults” OR “college student” OR “college students” OR undergraduate* OR “university student” OR “university students” OR “young adulthood” OR “new adulthood” OR “emerging adulthood”)

## CINAHL

(MH “Physical Activity” OR “physical activity” OR MH “Exercise” OR exercise OR MH “Walking” OR walking OR MH “Running” OR running OR MH “Cycling” OR cycling OR bicycling OR “active transport” OR MH “Sleep” OR sleep OR sleeping MH “Life Style, Sedentary” OR “sedentary behavior” OR sedentary OR MH “Sitting” OR sitting OR “physical inactivity” OR MH “Screen Time” OR “screen time” OR MH “Video Games” OR “video games” OR videogames OR video-games OR “video gaming” OR videogaming OR video-gaming OR gaming)

AND

(“ecological momentary assessment” OR “ecological momentary assessments” OR EMA OR mEMA OR “electronic diary” OR “electronic diaries” OR “ambulatory assessment” OR “ambulatory monitoring” OR “momentary assessment” OR “experience sampling method”)

AND

(MH “Young Adult” OR “young adult” OR “young adults” OR “emerging adult” OR “emerging adults” OR “new adult” OR “new adults” OR MH “Students, College” OR “college student” OR “college students” OR “university student” OR “university students” OR MH “Students, Undergraduate” OR undergraduate* OR “young adulthood” OR “new adulthood” OR “emerging adulthood”)
