# Supplementary material for: Use of Electronic Ecological Momentary Assessment Methodologies in Physical Activity, Sedentary Behavior, and Sleep Research in Young Adults: Systematic Review
Source: J Med Internet Res. 2023 Jun 29;25:e46783. doi: 10.2196/46783 (PMC10365632; doi:10.2196/46783)
Supplement: Multimedia Appendix 2 [file jmir_v25i1e46783_app2.docx]

Appendix 2.

Risk of bias assessment (*N* = 37)

| First Author  Last Name | Year | Q1 | Q2 | Q3 | Q4 | Q5 | Q6 | Q7 | Q8 | Q9 | Q10 | Q11 | Q12 | Q13 | Q14 | Overall Quality |
| --- | --- | --- | --- | --- | --- | --- | --- | --- | --- | --- | --- | --- | --- | --- | --- | --- |
| Andorko [48] | 2019 | Y | Y | N | Y | N | Y | Y | Y | Y | Y | Y | N | Y | Y | Good |
| Bedard [49] | 2017 | Y | Y | N | Y | N | Y | Y | Y | Y | Y | N | N | N | N | Fair |
| Bernstein [50] | 2019 | Y | Y | N | Y | Y | Y | Y | Y | Y | Y | N | N | Y | Y | Good |
| Bruening [42] | 2016 | Y | Y | N | Y | N | Y | Y | Y | Y | Y | Y | N | N | Y | Fair |
| Burke [51] | 2022 | Y | Y | N | Y | N | N | Y | Y | Y | Y | N | N | Y | Y | Fair |
| Das-Friebel [52] | 2020 | Y | Y | N | N | N | Y | Y | Y | Y | Y | Y | N | N | N | Fair |
| Gilchrist [53] | 2021 | Y | Y | N | N | N | N | Y | Y | Y | Y | Y | N | Y | Y | Fair |
| Kim [54] | 2015 | Y | Y | N | Y | N | N | Y | Y | Y | Y | Y | N | N | N | Fair |
| Kono [55] | 2022 | Y | Y | N | N | Y | N | Y | Y | Y | Y | Y | N | Y | Y | Fair |
| MacIntyre [56] | 2020 | Y | Y | N | Y | N | Y | Y | Y | Y | Y | N | N | Y | N | Fair |
| Maher [57] | 2020 | Y | Y | N | Y | Y | N | Y | Y | N | Y | Y | N | N | Y | Fair |
| Maher [58] | 2022 | Y | Y | N | Y | Y | Y | Y | Y | Y | Y | Y | N | Y | Y | Good |
| Marquet [43] | 2017 | Y | Y | N | Y | N | N | Y | Y | N | Y | N | N | N | N | Fair |
| Marquet [59] | 2018 | Y | Y | N | Y | N | N | Y | Y | Y | Y | N | N | N | Y | Fair |
| Mead [60] | 2022 | Y | Y | N | Y | Y | Y | Y | Y | Y | Y | Y | N | Y | Y | Good |
| Miller [61] | 2004 | Y | Y | N | N | N | Y | Y | Y | N | Y | Y | N | Y | Y | Fair |
| Milyavskaya – Study 1 [62] | 2018 | Y | Y | N | Y | N | Y | Y | Y | Y | Y | N | N | N | Y | Fair |
| Nadell [44] | 2015 | Y | Y | N | Y | N | N | Y | Y | Y | N | N | N | N | Y | Fair |
| Parsons [63] | 2022 | Y | Y | N | Y | N | Y | Y | Y | Y | Y | N | N | N | Y | Fair |
| Ponnada [45] | 2022 | Y | Y | N | N | N | Y | Y | Y | N | Y | Y | N | N | N | Fair |
| Romanzini [64] | 2019 | Y | Y | N | N | N | N | Y | Y | N | Y | Y | N | Y | N | Fair |
| Runyan [65] | 2013 | Y | Y | N | Y | N | Y | Y | Y | N | Y | N | N | N | Y | Fair |
| Sala [66] | 2017 | Y | Y | N | N | N | Y | Y | Y | Y | Y | N | N | N | N | Fair |
| Sano [67] | 2018 | Y | Y | N | N | N | N | Y | Y | Y | N | N | N | Y | N | Fair |
| Shah [68] | 2021 | Y | Y | N | Y | N | Y | Y | Y | N | Y | Y | N | Y | N | Fair |
| Sladek [69] | 2020 | Y | Y | N | N | N | Y | Y | Y | Y | Y | Y | N | N | Y | Fair |
| Sperry [70] | 2018 | Y | Y | N | Y | N | Y | Y | Y | Y | Y | N | N | Y | N | Fair |
| Sperry [71] | 2022 | Y | Y | N | Y | N | Y | Y | Y | Y | Y | Y | N | N | N | Fair |
| Strahler [46] | 2016 | Y | Y | N | Y | N | Y | Y | Y | Y | Y | Y | N | N | Y | Fair |
| Titone [72] | 2022 | Y | Y | N | Y | N | Y | Y | Y | Y | Y | Y | N | N | Y | Fair |
| Von Haaren [73] | 2013 | Y | Y | N | N | N | Y | Y | Y | Y | Y | Y | N | N | N | Fair |
| Von Haaren [74] | 2016 | Y | Y | N | N | N | Y | Y | Y | Y | Y | Y | N | Y | N | Fair |
| Van Woerden [47] | 2022 | Y | Y | N | N | N | N | Y | Y | N | Y | N | N | N | Y | Fair |
| Walter [75] | 2013 | Y | Y | N | Y | N | Y | Y | Y | N | Y | Y | N | N | Y | Fair |
| Wen [76] | 2022 | Y | Y | N | N | N | Y | Y | Y | N | Y | N | N | Y | N | Fair |
| Wu [77] | 2021 | N | Y | N | N | N | Y | Y | Y | N | Y | Y | N | N | N | Fair |
| Yap [78] | 2022 | Y | Y | N | Y | Y | Y | Y | Y | Y | Y | Y | N | N | Y | Good |

*Note.* Questions from the National Heart Lung and Blood Institute Quality Assessment Tool for Observational Cohort and Cross-sectional Studies [38]*.* Y indicates “Yes”; N indicates “No or Other (cannot determine).”

Q1. Was the research question or objective in this paper clearly stated?

Q2. Was the study population clearly specified and defined?
Q3. Was the participation rate of eligible persons at least 50%?
Q4. Were all subjects selected or recruited from the same or similar populations (including same time period)? Were inclusion/exclusion criteria for being in the study prespecified and applied uniformly to all participants?
Q5. Was sample size justification, power description, or variance and effect estimates provided?

Q6. For the analyses in this paper, were the exposure(s) of interest measured prior to the outcome(s) being measured?

Q7. Was the timeframe sufficient so that one could reasonably expect to see an association between exposure and outcome if it existed?

Q8. For exposures that can vary in amount or level, did the study examine different levels of the exposure as related to the outcome (e.g., categories of exposure, or exposure measured as continuous variable)?

Q9. Were the exposure measures (independent variables) clearly defined, valid, reliable, and implemented consistently across all study participants?

Q10. Was the exposure(s) assessed more than once over time?

Q11. Were the outcome measures (dependent variables) clearly defined, valid, reliable, and implemented consistently across all study participants?

Q12. Were the outcome assessors blinded to the exposure status of participants?

Q13. Was loss to follow-up after baseline 20% or less?

Q14. Were key potential confounding variables measured and adjusted statistically for their impact on the relationship between exposure(s) and outcome(s)?

Overall Quality [39]: poor (0–4), fair (5–10), or good (11-14)
