# Supplementary material for: Use of Electronic Ecological Momentary Assessment Methodologies in Physical Activity, Sedentary Behavior, and Sleep Research in Young Adults: Systematic Review
Source: J Med Internet Res. 2023 Jun 29;25:e46783. doi: 10.2196/46783 (PMC10365632; doi:10.2196/46783)
Supplement: Multimedia Appendix 3 [file jmir_v25i1e46783_app3.docx]

Appendix 3.

Outcomes, psychometric support, and compliance

| Author | Movement Behaviors | Other Outcomes Measured via e-EMA | Psychometric Support for  e-EMA Measures | Attrition Across Days or Waves | Prompts Delivered | Latency (mean time) | Response Rate | Missing Data |
| --- | --- | --- | --- | --- | --- | --- | --- | --- |
| Andorko et al, 2019 [48] | PA^a^  SB^a^ |  | Modified from a validated scale | N^f^ | N | N | 82.51% | Participants completing less than 75% were excluded from analysis.^e^ |
| Bedard et al, 2017 [49] | PA ^b^ | Context (activity, location, social company), PA related outcomes (outcome expectancy, barrier self-efficacy, intentions), affective states, self-control | Validated items, items from a validated scale, and newly created items | N | N | N | 56% | 36% excluded from analysis due to responding fewer than 2 prompts per day on fewer than 5 days.^e^ |
| Bernstein et al, 2019 [50] | PA^a^ | Anxiety, sadness, cheerfulness, and contentment (emotional inertia, variability, and instability) | Newly created items | N | N | N | N | N |
| Bruening et al, 2016 [42] | PA^a^  SB^a^ | Eating and drinking, current activity including PA and SB | Modified from validated scale and newly created items | N | N | 6.90 - 7.25 min. | N | 16% PA data excluded due to missingness.^e^ |
| Burke et al, 2022 [51] | Sleep ^b, c^ | Non-suicidal self-injury urge severity,  negative affect | Newly created items | N | N | N | N | 4.86% missing data (not specific to e-EMA).^e^ |
| Das-Friebel et al, 2020 [52] | Sleep ^b, c^ | Affect (positive and negative) | Modified from validated scale; Reliability from current study reported | N | N | N | N | 3% excluded due to response rate < 60%. Instructed to ignore signals delivered during incompatible activity.^e^ |
| Gilchrist et al, 2021 [53] | PA^a^ | Body surveillance during PA,  affective judgments of PA (pleasure, enjoyment) | Modified from validated scales and newly created items; Reliability from current study reported | N | N | 7.33 min. | 43 prompts per person | Excluded for response rate < 50% (*n* = 4), technical errors (*n* = 1). Instructed to ignore signals delivered during incompatible activity. ^e^ |
| Kim et al, 2015 [54] | PA ^b^ | Fatigue, depressive mood, anxious mood | Modified from validated scales and newly created items | N | N | N | 92% | 37% excluded due to attrition or technical errors.^e^ |
| Kono, et al, 2022 [55] | PA ^a^  Sleep ^a^ | Leisure time activities including PA and sleep/resting, 8 daily experiences (freedom, intrinsic motivation, enjoyment, effort, stimulation, comfort, Shiawase, Ikigai) | Previously used items, validated items, and newly created items. Reliability from current study reported. | N | N | 97 sec. | 54.4% | N |
| MacIntyre et al, 2020 [56] | PA ^b, c^ | Body comparisons,  body dissatisfaction, thoughts of exercising | Modified from validated scales and newly created items | N | N | N | 81.8% | Excluded for compliance < 20% (*n* = 2). Expectation Maximization Imputation to address missing of < 15%.^e^ |
| Maher et al,  2020 [57] | PA ^b^ | Diet (food/fluids),  health behavior engagement,  feelings (stress, hunger, and thirst) | Modified from validated scales. Previously used items. | N | N | N | 85% | Potential missing due to gaps in prompting protocol. Compliance did not vary based on time of day, weekend/day, steps taken, sex, or body mass index. |
| Maher et al, 2022 [58] | PA ^b^  Sleep ^b, c^ | Stress | Previously used items. | N | 1,654 | N | 85%;  24.6% - 100% per participant | Low missing for stress EMA because of aggregation within day. |
| Marquet et al, 2017 [43] | PA ^a, b, c^ | Pokemon GO behaviors (location, social company, transportation, environment) | Newly created items | N | N | N | N | Excluded because missing first online survey or had insufficient EMA data (*n* = 49). Excluded because < 80% compliance (*n* = 7).^e^ |
| Marquet et al, 2018 [59] | PA ^a, b, c^ | Pokemon GO playing behaviors (playing time, playing environment, and playing conditions such as social or individual play) | Newly created items | N | N | N | Average per participant: 4.4 (weekdays);  2 (weekend days) | Excluded because < 80% compliance (*n* = 7). Missing data were imputed using predictive mean matching.^e^ |
| Mead et al, 2022 [60] | Sleep ^b, c^ | Theory of planned behavior constructs related to sleep opportunity: attitudes, subjective norms, perceived behavioral control, and intention to sleep | Modified from validated scale. | N | N | N | 92% | Excluded participant-days with missing >50% EMA (*n*=2), participants missing >50% of EMA days (*n* = 1), and timing errors (25 total signals).^e^ |
| Miller et al, 2004 [61] | PA ^c^  Sleep ^c^ | Stress | Newly created items. | N | N | N | N | 13% of entries excluded due to responding outside of 1 hr window.^e^ |
| Milyavskaya et al, 2018 Study 1 [62] | PA ^a^  Sleep ^a^ | Fear of missing out,  current activity including PA and sleep | Newly created items. Reliability reported. | N | N | N | Daytime: 68%  End of day/night: 90% | 32% missing data. Excluded participants due to response rates < 20% (*n* = 19). Lower missing during day than on night-time prompts. |
| Nadell et al, 2015 [44] | PA ^c^ | Smoking level and urges | Newly created items. | N | N | N | N | 83% had usable data for Days 2 - 7; Non-usable data were due to fewer days carrying device or data were not collected on certain days. |
| Parsons et al, 2022 [63] | Sleep ^c^ | Emotion intensity, duration, and regulation strategies | Modified from validated scales and newly created items. Validity and reliability reported in current study. | N | N | N | 72.8% | Excluded for < 20% completed surveys (*n* = 6). Listwise deletion for individual assessments.^e^ |
| Ponnada et al, 2022 [45] | PA ^a, b^  SB ^a, b^ | Core-constructs (internal and external factors, reflective processes, and reactive processes related to behavior adoption and maintenance),  person-level characteristics | Newly created items. | N | μEMA only: 662,397 (83.81%) | μEMA only:  4.8 sec. | μEMA only: 67.7% | Students reported not wanting to respond during school exams. Patterns not reported for main study.^e^ |
| Romanzini et al, 2019 [64] | SB ^a, b^ | Physical position, location, and activity, social company | Newly created items. Validity reported for current study. | N | N | N | Greater than 50% had response rate > 60%. | Excluded 19% of data due to mismatched mEMA and accelerometer data. Excluded 15% of prompts due to difficulty categorizing “Other” responses. Instructed to ignore signals delivered during incompatible activity.^e^ |
| Runyan et al, 2013 [65] | PA ^a, c^ | Current activity including PA, social interactions, studying, etc. | Newly created items. | Y | N | N | Daytime: 40.1% (Week 1)   19.1% (Week 3) | Some participants reported missing surveys due to not hearing the alarm.^e^ |
| Sala et al, 2017 [66] | PA ^a^ | Stress | Validated subscale. Newly created item. Reliability reported for current study. | N | N | N | 66%  14% – 96% per participant | N |
| Sano et al, 2018 [67] | PA ^a, b^  Sleep ^a, b^ | Activities including PA and sleep, social interactions, caffeine intake, alcohol intake, drug intake, overall health condition, mood, stress | Newly created items | Y | N | N | 92% - 97% average per day | N |
| Shah et al, 2021 [68] | PA ^b^  Sleep ^b^ | Depressive and anxious moods, stress,  diet | Newly created items | N | N | N | 77.5% per participant | Multivariate imputation to address missing data.^e^ |
| Sladek et al, 2020 [69] | Sleep ^b, c^ | Stress, potential covariates (caffeine intake, eating, negative affect) | Previously used items. | N | N | N | 94.7% | N |
| Sperry et al, 2018 [70] | PA ^a^ | Behaviors (posture, PA, talking, social activity, eating, drinking, caffeine intake, nicotine) | Newly created items. | N | N | N | 72%; 4 – 12 per participant | N |
| Sperry et al, 2022 [71] | Sleep ^c^ | Affect (negative and positive), cognition (difficulty concentrating, racing thoughts), behavior characteristics (impulsivity) | Newly created items. Reliability reported for current study. | N | N | N | N | Excluded for completing < 20 ESM questionnaires per week (*n* = 71); Imputation based in Bayesian Markov chain Monte Carlo estimation.^e^ |
| Strahler et al, 2016 [46] | PA ^b, c^ | Fatigue | Previously used item. | N | N | N | N | Missing data were excluded listwise.^e^ |
| Titone et al, 2020 [72] | Sleep ^b^ | Hypomanic symptoms (happy, self-confident, needing less sleep, talkative), depressive symptoms (sad, hopeless, low self-esteem, low energy) | Modified from validated scales. | N | N | N | N | 72% usable observations, not solely EMA data. Listwise deletion and Full Information Maximum Likelihood (sensitivity analysis) to address missing data.^e^ |
| von Haaren et al, 2013 [73] | PA ^b^ | Affect (energetic arousal, valence, and calmness) | Validated scale for EMA use. | N | N | N | N | Missing data attributed to non-wear time and variable waking times. |
| von Haaren et al, 2016 [74] | PA ^b, d^ | Stress | Previously used items. | N | N | N | N | N |
| Van Woerden et al, 2022 [47] | PA ^a^ | Activities including PA, eating, drinking, or none of these.  Social context with passive time and location | Newly created items. | Y | N | < 6 mins | 37 per participant | Examined compliance by demographic characteristics, prompting protocol, and phone operating systems. Lower compliance before midday, on weekends, and after initial wave. |
| Walter et al, 2013 [75] | PA ^b^ | Affect (energetic arousal, valence, and calmness) | Validated scale for EMA use. | Y | N | N | 72% at Time 0  38% at Time 4 | High number of missing data for mood prior to and immediately after training. |
| Wen et al, 2022 [76] | PA ^b^  Sleep ^b^ | Affect (positive: happiness, relaxation, activeness, interest; negative: anxiety, depression, anger, bore, sadness) inertia and variability | Newly created items. Reliability reported for current study. | N | N | N | N | N |
| Wu et al,  2021 [77] | PA ^b^  Sleep ^b^ | Context, current activity, mood (sadness, loneliness, contentment, stress, energy level) | Newly created items. | Y | N | N | N | Missing data were *scattered throughout*.^e^ |
| Yap et al, 2022 [78] | Sleep ^c, d^ | Stress | Previously used items. Reliability reported for current study. | N | N | N | N | N |

*Note.* When psychometric support was not discussed, it was assumed the items were newly created for the study.

^a^ data collection via e-EMA

^b^ data collection via accelerometer (or pedometer)

^c^ data collection via recall survey (daily, weekly, or longer duration) or diary

^d^ data collection via other method
^e^ Examination of specific patterns of missing data/low compliance were not discussed.
^f^ N: No or not reported
